# Effects of Wii-Fit games training on motor skills, balance, and agility in children with developmental coordination disorder: a systematic review and meta-analysis

**DOI:** 10.1186/s13102-025-01384-z

**Published:** 2025-11-11

**Authors:** Jindong Chang, Zhuoling Lei, Pengwei Ma, Juan Cui, Wenbing Zhu

**Affiliations:** 1 School of Physical Education, Southwest University, Chongqing, 400715 China; 2 College of Physical Education and Health Science, Chongqing Normal University, Chongqing, 401331 China; 3 Institute of Motor Quotient, Southwest University, Chongqing, 400715 China; 4 Faculty of Sports and Exercise Science, University of Malaya, Kuala Lumpur, 50603 Malaysia

**Keywords:** Wii-Fit games, DCD, Motor skills, Balance, Meta-Analysis

## Abstract

**Objective:**

The purpose of this study was to systematically investigate the effects of Wii-Fit game training on children with developmental coordination disorder (DCD), specifically elucidating its impact on enhancing motor skills, balance, and agility capabilities.

**Methods:**

The literature search was conducted from March 22 to June 12, 2025, encompassing the PubMed, Embase, Web of Science, and Cochrane Library databases. We systematically searched for randomized controlled trials (RCTs) published in databases from their inception through June 12, 2025, using predefined inclusion and exclusion criteria to perform study selection. The methodological quality (risk of bias 2.0) of the included studies was assessed using the Cochrane Risk of Bias tool. Statistical analyses were performed using RevMan software (version 5.4.1). Publication bias was evaluated using Stata software (version 16.0).

**Results:**

This systematic review and meta-analysis included six RCTs with a total of 162 participants. The meta-analysis revealed that Wii-Fit game training may lead to improvement in motor skills (SMD = 0.54, 95% CI [0.06, 1.02], *p* = 0.03) and appears beneficial for balance (SMD = 0.69, 95% CI [0.36, 1.01], *p* < 0.0001). For agility, the effect size was borderline significant (SMD = 0.65, 95% CI [0.00, 1.30], *p* = 0.05), suggesting only a possible positive trend.

**Conclusion:**

Wii-Fit training may improve motor skills and balance in children with DCD, with a potential but inconclusive effect on agility. These results should be interpreted with caution due to small sample sizes, variability in outcome measures, and moderate methodological quality across the included studies. Further large-scale, rigorously designed RCTs are needed to confirm these preliminary findings. The study protocol was prospectively registered with PROSPERO (CRD420251073789).

**Supplementary Information:**

The online version contains supplementary material available at 10.1186/s13102-025-01384-z.

## Introduction

Developmental Coordination Disorder (DCD) is characterized by significant deficits in motor skill that can interfere with daily activities and academic performance [1]. Epidemiological studies indicate that the prevalence of DCD is between 5% and 6%, with a higher incidence among school-aged males [2]. DCD manifests as various motor dysfunctions, including impairments in fine and gross motor coordination, balance control, and movement disorders [3]. Research on the integration of upper limb motor control has revealed that these motor deficits are present in both the dominant and non-dominant limbs, occurring in both unilateral and bilateral tasks [4].

In addition to motor coordination deficits, children with DCD often exhibit impairments in dynamic balance strategies compared to typically developing peers, and their reduced participation in physical activity may contribute to secondary issues such as lower cardiopulmonary fitness [5, 6]. Beyond motor skills, they are also at risk of difficulties in executive functions, visual perception, attention, and social participation, which can negatively affect quality of life and overall development [7–9]. Therefore, comprehensive assessment and intervention targeting core motor deficits and functional impairments are critical.

Physical or occupational therapy is one of the traditional approaches for DCD, primarily utilizing repetitive training to enhance motor skills and fine coordination [10]. However, such repetitive formats may be less engaging for children, prompting interest in more interactive approaches such as exergaming [11]. In recent years, exergaming (exercise-based gaming) has emerged as a novel intervention method for rehabilitating children with DCD. Motion-based platforms, such as Wii-Fit and Kinect, have been shown to improve motor performance and fitness while providing a more engaging therapeutic environment [2, 12]. Evidence also suggests positive effects on cognitive functions (e.g., attention, executive function) and social interaction through multiplayer gaming, further broadening its rehabilitation value [7–9]. Different exergaming devices vary in training intensity. For instance, Kinect, which utilizes full-body motion capture technology, provides higher-intensity training, whereas Wii-Fit demonstrates more pronounced effects in improving fine motor skills in children with DCD [2, 12]. The application of VR (virtual reality) technology further expands the possibilities for motor interventions. Studies have confirmed that VR-based exercise programs can enhance cardiopulmonary endurance, increase participation in physical activities, and boost intrinsic motivation in children with DCD [6]. The findings of this study also highlight the need to develop more tailored and effective rehabilitation plans based on the individual needs of children with DCD.

Wii-Fit represents an innovative exercise training modality with unique therapeutic mechanisms for motor interventions in children with DCD [13]. Although Wii-Fit represents an early example of motion-based gaming and has been widely studied, newer technologies (e.g., Kinect, Oculus-based VR) now offer more sophisticated platforms for motor rehabilitation. However, Wii-Fit remains accessible and may still hold clinical value due to its affordability and evidence base. Wii-Fit employs motion-based, screen-mediated gaming technology, which differs from fully immersive virtual reality (VR) systems. While not immersive, its real-time feedback and goal-oriented tasks may enhance motor learning through engagement [14]. Studies indicate that Wii-Fit training can enhance balance strategies, visual perception, and executive functions, though evidence on long-term effectiveness remains limited, with high attrition rates reported in follow-ups [5, 7, 15]. Comparative findings between Wii-Fit and Kinect remain mixed, with some studies reporting similar improvements and others highlighting the higher training intensity achievable with Kinect [2, 12]. Controversy also exists regarding Wii-Fit’s impact on motor learning rates in DCD. Although some studies report significantly slower motor learning rates in DCD children compared to typically developing peers [16], others found no significant differences in retention and transfer capabilities [17]. These discrepancies underscore the need for more systematic investigations.

## Methods

This study adhered to the Preferred Reporting Items for Systematic Reviews and Meta-Analyses (PRISMA) statement guidelines [18]. The study protocol was prospectively registered with PROSPERO (Registration number: CRD420251073789). No deviations from the registered protocol were made during the study implementation.

### Literature search

The systematic literature search was conducted from March 22 to June 12, 2025, spanning PubMed, Embase, Web of Science, and the Cochrane Library. We employed a comprehensive search strategy combining Medical Subject Headings (MeSH) and free-text keywords, including “developmental coordination disorder,” “motor skills,” “balance,” “children,” and “virtual reality”, using Boolean operators (AND/OR) to identify randomized controlled trials published from database inception through June 12, 2025. Initial electronic search results underwent iterative screening rounds, supplemented by manual reference tracking of included studies and related literature to identify additional eligible publications. Only peer-reviewed literature published in English was included; grey literature was systematically excluded. Detailed records are shown in Table 1.

### Inclusion and exclusion criteria

The selection criteria for this study were as follows: (1) Subjects: children under 12 years of age with a clinical diagnosis of DCD; (2) Study design: randomized controlled trial (RCTs); (3) Type of intervention: Wii-Fit game training; Results: The children’s Movement Assessment Battery for Children—Second Edition (MABC-2) and Bruininks-Oseretsky Motor Ability Test version 2 (BOT-2) were used to evaluate their motor skills, balance ability, and agility. Non-randomized controlled studies and repeated published studies were excluded; (5) Language: only studies published in English were included; (6) Literature type: only peer-reviewed journal articles were included; Conference abstracts, unpublished reports, and dissertations were excluded. Exclusion criteria: (1) non-randomized controlled trials; (2) Duplicate publications; (3) lack of adequate outcome data; Or (4) the intervention or assessment tool is not clearly described.

### Study selection

The two independent reviewers independently screened the titles and abstracts of all retrieved records based on the predefined inclusion and exclusion criteria. Full-text articles were obtained for studies that appeared to meet the eligibility criteria or where eligibility was unclear. Although the reviewers were not blinded to the study authors or journals, efforts were made to minimize selection bias through independent and standardized screening. Discrepancies between two independent reviewers in the implementation process were resolved with the involvement of the corresponding author.

**Table 1:** Search strategy

Databases	Search terms
PubMed	(“Motor Skills Disorders“[MeSH Terms] OR (“developmental coordination disorder“[Title/Abstract] OR “coordination disorder“[Title/Abstract] OR “developmental coordination disorders“[Title/Abstract])) AND (“Exergaming“[MeSH Terms] OR (“virtual reality“[Title/Abstract] OR “video game“[Title/Abstract] OR “computer game“[Title/Abstract] OR “digital game“[Title/Abstract] OR “exergam“[Title/Abstract] OR “serious game“[Title/Abstract] OR “augmented reality“[Title/Abstract] OR “interactive game“[Title/Abstract] OR “motion-based“[Title/Abstract] OR “nintendo wii“[Title/Abstract] OR “xbox kinect“[Title/Abstract] OR “PlayStation“[Title/Abstract])) AND (“Motor Skills“[MeSH Terms] OR (“balance“[Title/Abstract] OR “motor competence“[Title/Abstract] OR “gross motor“[Title/Abstract] OR “fine motor“[Title/Abstract] OR “motor development“[Title/Abstract]))
Embase	‘motor skills disorders’:ab, ti OR ‘developmental coordination disorder’:ab, ti OR ‘coordination disorder’:ab, ti OR ‘developmental coordination disorders’:ab, ti ‘virtual reality’:ab, ti OR exergaming: ab, ti OR ‘video game’:ab, ti OR ‘computer game’:ab, ti OR ‘digital game’:ab, ti OR exergam: ab, ti OR ‘serious game’:ab, ti OR ‘augmented reality’:ab, ti OR ‘interactive game’:ab, ti OR ‘motion based’:ab, ti OR ‘nintendo wii’:ab, ti OR ‘xbox kinect’:ab, ti OR playstation: ab, ti ‘motor skills’:ab, ti OR balance: ab, ti OR ‘motor competence’:ab, ti OR ‘gross motor’:ab, ti OR ‘fine motor’:ab, ti OR ‘motor development’:ab, ti
Web of Science	(((TS=(Motor Skills Disorders)) OR TS=(developmental coordination disorder)) OR TS=(coordination disorder)) OR TS=(developmental coordination disorders) and Preprint Citation Index (Exclude – Database) ((((((((((((TS=(Virtual Reality)) OR TS=(Exergaming)) OR TS=(video game)) OR TS=(computer game)) OR TS=(digital game)) OR TS=(exergam)) OR TS=(serious game)) OR TS=(augmented reality)) OR TS=(interactive game)) OR TS=(motion-based)) OR TS=(nintendo wii)) OR TS=(xbox kinect)) OR TS=(PlayStation) and Preprint Citation Index (Exclude – Database) (((((TS=(Motor Skills)) OR TS=(balance)) OR TS=(motor competence)) OR TS=(gross motor)) OR TS=(fine motor)) OR TS=(motor development) and Preprint Citation Index (Exclude – Database)
Cochrane	Motor Skills Disorders OR developmental coordination disorder OR coordination disorder OR developmental coordination disorders Virtual Reality OR Exergaming OR video game OR computer game OR digital game OR exergam OR serious game OR augmented reality OR interactive game OR motion-based OR nintendo wii OR xbox kinect OR PlayStation Motor Skills OR balance OR motor competence OR gross motor OR fine motor OR motor development

### Data extraction

The two reviewers independently conducted the data extraction process using a standardized data extraction form. The following details were meticulously collected: (1) fundamental study information, which encompassed the author’s name, year of publication, country of origin, trial design, and participant characteristics; (2) experimental characteristics, including the type of exercise, duration, frequency, and intervention cycle; (3) outcome measures.

### Risk of bias and quality of evidence

Three researchers evaluated and graded the studies based on the Cochrane Risk of Bias 2.0 tool [19]. The quality of evidence was rated using the GRADE system for assessment, development, and evaluation [20].This assessment was independently conducted by two researchers, with a third researcher consulted in cases of disagreement to reach a consensus.

### Data analysis and synthesis

The statistical analysis was conducted utilizing RevMan 5.4.1 software. Continuous variables were articulated as a standardized mean difference (SMD) accompanied by 95% confidence intervals (CI). The heterogeneity of the data was evaluated using the p-value and I² statistic. High heterogeneity was denoted by a p-value less than 0.01 and an I² greater than 50%, in which case a random-effects model was employed [21]. Conversely, low heterogeneity, indicated by a p-value >0.01 and an I² < 50%, warranted using a fixed-effects model for the meta-analysis [22]. The potential for publication bias was assessed using Egger’s regression test and funnel plots, conducted in Stata 16.0 software.

## Results

### Search results

**Fig. 1:**
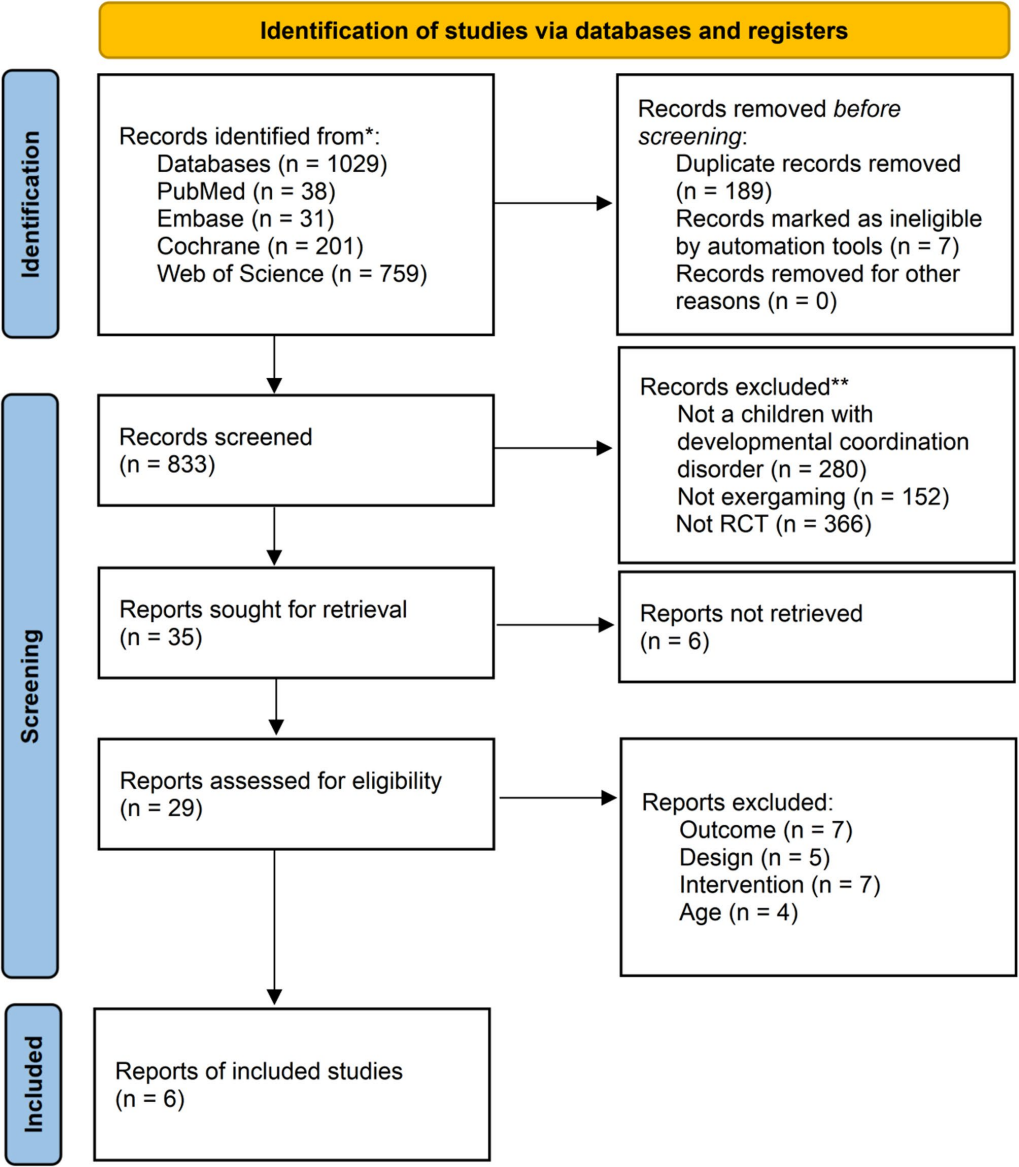
PRISMA flowchart of included and excluded studies

Figure 1 illustrates the literature screening workflow. Initial database searches identified 1,029 records. Following removal of duplicates and irrelevant entries, 833 records underwent systematic screening. Titles and abstracts were evaluated against eligibility criteria, with subsequent full-text assessment of potentially relevant studies. This process yielded six articles meeting inclusion criteria for quantitative synthesis.

### Characteristics of the included studies

The characteristics of the included studies are summarized in Table 2. Six randomized controlled trials, involving a total of 162 DCD patients with a mean age of 9 years, were included in this analysis. These studies originated from three countries: Brazil [15], the United Kingdom [23, 24], and the Netherlands [12, 25, 26]. All studies clearly defined participant inclusion and exclusion criteria based on a DCD diagnosis.

Interventions primarily utilized Wii-Fit-based game training. Two studies compared Wii-Fit games to Xbox Kinect games [12, 15], while four compared the Wii-Fit intervention to a usual activity control group [23–26]. The intervention frequency ranged from 1 to 3 sessions per week, each lasting 10 to 30 min, over a duration of 4 to 6 weeks. Control groups engaged in either routine activities or Xbox Kinect game training.

Meta-analysis was performed to assess outcomes in motor skills, balance, and agility. These domains were evaluated using either the BOT-2 or MABC-2 assessment instruments across the studies. Specifically, MABC-2 was employed in five studies, and BOT-2 was included in four studies in the meta-analysis.

### Risk of bias and quality of evidence

**Fig. 2:**
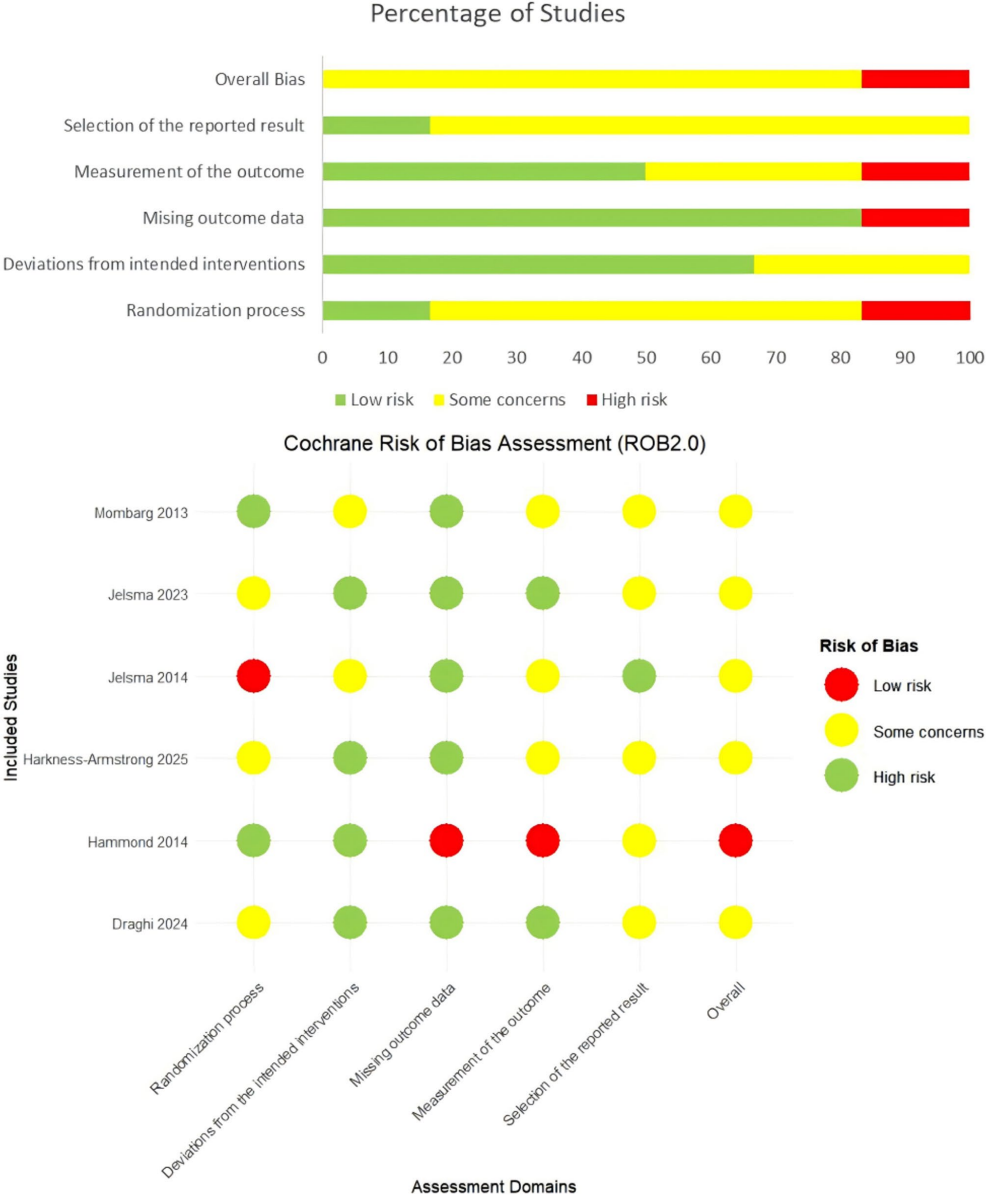
Risk of bias assessment for included studies

The Cochrane Risk of Bias tool (ROB 2.0) was used to evaluate the quality of the included six RCTs. The results in Fig. 2 indicate that the overall quality of a study was relatively high and rated as “low risk”. In contrast, some studies had problems with the randomization process, outcome measures, and other aspects, and some studies were even rated as “high risk,” which may be a source of heterogeneity across studies. The GRADE system was used to grade the overall quality of evidence. The detailed results are shown in Table 3. According to GRADE, the overall quality of evidence was moderate. However, due to the limitations of study quality and the number of included studies, caution is needed in interpreting the conclusions.

**Table 2:** Characteristics of the inclusion studies

ID	Study	Country	Sample size (E/C)	Gender (F/M)	Age(years)	Intervention arm	Time (min)	Frequency (times/ week)	Intervention duration (weeks)	Control arm	Instruments
1	Draghi 2024	Brazil	17/19	EG:7/10 CG: 9/10	EG:9.06 ± 1.25 CG:9.00 ± 1.05	Training was based on Wii-Fit games	20	1–2	5 weeks (9 times)	Training was based on Xbox Kinect games	MABC-2
2	Hammond 2014	UK	10/8	EG: 2/8 CG:2/6	EG:8.53 ± 1.15 CG:9.53 ± 1.42	Training was based on Wii-Fit games	10	3	4 weeks	Treatment as usual	BOT-2
3	Harkness-Armstrong 2025	UK	11/6	EG: 3/8 CG:2/4	9 ± 1	Training was based on Wii-Fit games	≥ 30	3	6 weeks (≥ 15 times)	Treatment as usual	MABC-2
4	Jelsma 2014	Netherlands	14/14	EG: 5/9 CG:5/9	EG:8.73 ± 1.42 CG:7.67 ± 1.18	Training was based on Wii-Fit games	30	3	6 weeks	Treatment as usual	1.MABC-2 2.BOT-2
5	Jelsma 2023	Netherlands	17/17	EG: 8/9 CG:6/11	EG:9.1 ± 1 CG:8.9 ± 1.2	Training was based on Wii-Fit games	20	1–2	5 weeks (9 times)	Training was based on Xbox Kinect games	1.MABC-2 2.BOT-2
6	Mombarg 2013	Netherlands	15/14	EG: 3/12 CG:3/11	EG:9.5 ± 1.8 CG:9.7 ± 1.11	Training was based on Wii-Fit games	25–30	3	6 weeks	Treatment as usual	1.MABC-2 2.BOT-2

MABC-2=Movement Assessment Battery for Children—Second Edition; BOT-2༝Bruininks-Oseretsky Test of Motor Proficiency Second Edition; “-”༝not reported. EG: experimental group; CG: control group.

**Table 3:** Quality of evidence (GRADE Assessment)

ID	Study	Study design	Risk of bias	Inconsistency	Indirectness	Imprecision	Other considerations	Certainty	Importance
1	Draghi 2024	RCT	not serious	not serious	not serious	not serious	none	⨁⨁⨁⨁ High	important
2	Hammond 2014	RCT	very serious	not serious	not serious	not serious	none	⨁⨁◯◯ Low	important
3	Harkness-Armstrong 2025	RCT	serious	not serious	not serious	not serious	none	⨁⨁⨁◯ Moderate	important
4	Jelsma 2014	RCT	serious	not serious	not serious	not serious	none	⨁⨁⨁◯ Moderate	important
5	Jelsma 2023	RCT	not serious	not serious	not serious	not serious	none	⨁⨁⨁⨁ High	important
6	Mombarg 2013	RCT	serious	not serious	not serious	not serious	none	⨁⨁⨁◯ Moderate	important

**Table 4:** Effects of Wii-Fit game training compared with control in children with DCD in the meta-analysis

Variables	Control group	Study	SMD	95% CI	Weight (%)	Individual study *P* values	I² (%)	*P*
**Motor skills**	Treatment as usual	Hammond 2014	1.01	0.01, 2.01	17.2	0.05	0	0.19
		Jelsma 2014	1.08	0.28, 1.89	23.5	0.008		
	Xbox Kinect games	Draghi 2024	0.16	−0.50, 0.82	30.1	0.63		
		Jelsma 2023	0.22	−0.45, 0.90	29.2	0.52		
**Balance**	Treatment as usual	Hammond 2014	1.07	0.06, 2.08	10.2	0.04	0	0.80
		Harkness-Armstrong 2025	1.11	0.03, 2.20	8.8	0.04		
		Jelsma 2014	0.75	−0.02, 1.52	17.4	0.06		
		Mombarg 2013	0.82	0.05, 1.58	17.8	0.04		
	Xbox Kinect games	Draghi 2024	0.38	−0.28, 1.05	23.7	0.25		
		Jelsma 2023	0.51	−0.18, 1.19	22.1	0.14		
**Agility**	Treatment as usual	Hammond 2014	0.37	−0.57, 1.31	28.9	0.44	46	0.16
		Jelsma 2014	1.31	0.48, 2.14	33.4	0.002		
		Mombarg 2013	0.29	−0.44, 1.02	37.8	0.44		

### Meta-Analysis

#### Effects of Wii-Fit games training on motor skills in children with DCD

**Fig. 3:**
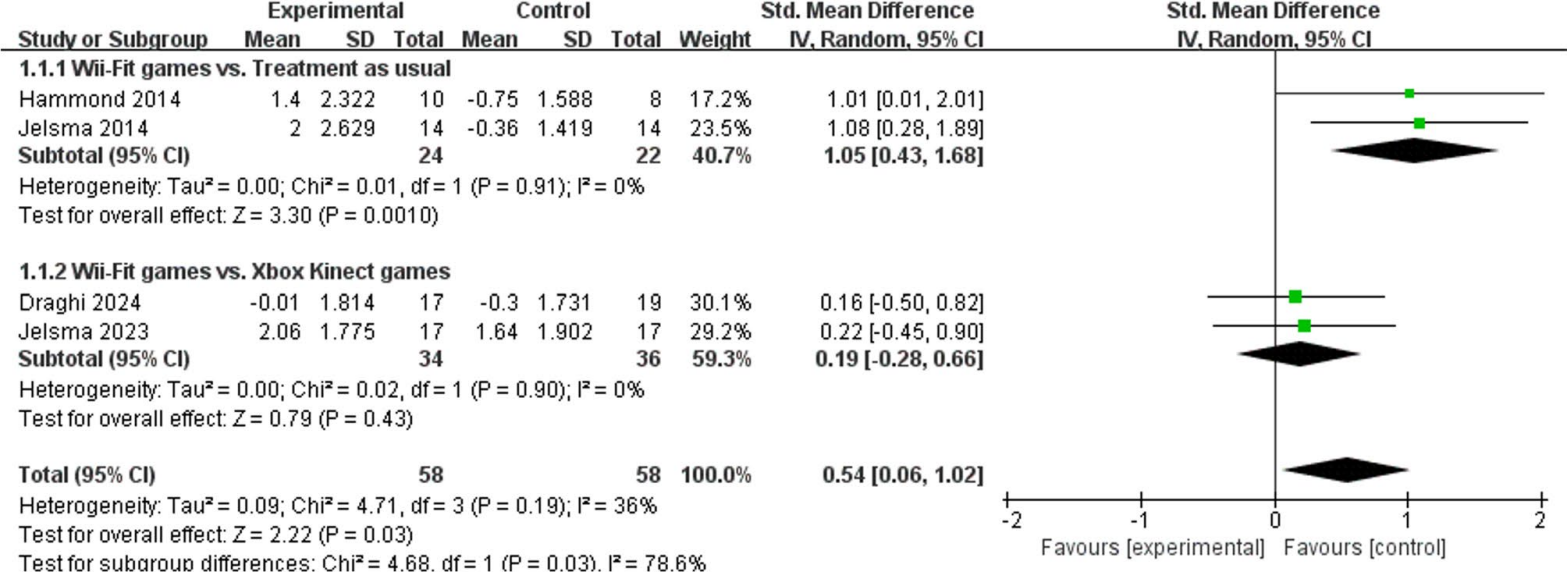
Forest plot of the effect of Wii-Fit game training on motor skills.

Note: Values to the right favor the physical exercise (experimental) group

Four studies (*n* = 116) evaluated the effects of Wii-Fit game training on motor skills in children with DCD [12, 15, 23, 25]. As shown in Fig. 3, the meta-analysis revealed a statistically significant improvement in motor skills favoring the Wii-Fit group over the control group (SMD = 0.54, 95% CI [0.06, 1.02], *p* = 0.03), with low heterogeneity (I² = 36%, *p* = 0.19), indicating consistency across studies. Subgroup analyses were performed to compare Wii-Fit game training against different control conditions: (1) Wii-Fit vs. Usual activity: This subgroup showed a large and statistically significant effect favoring Wii-Fit (SMD = 1.05, 95% CI [0.43, 1.68], *p* < 0.001), with no significant heterogeneity (I² = 0%, *p* = 0.91); (2) Wii-Fit vs. Xbox Kinect Game Training: No statistically significant difference was found between the two active interventions (SMD = 0.19, 95% CI [−0.16, 0.54], *p* = 0.43), also with no significant heterogeneity (I² = 0%, *p* = 0.90). These results suggest that Wii-Fit game training significantly improves motor skills in children with DCD compared to usual activity. However, when compared directly to Xbox Kinect game training, Wii-Fit demonstrated no statistically significant superiority, indicating comparable effects between the two game-based interventions. Detailed records are shown in Table 4.

### Effects of Wii-Fit games training on balance in children with DCD

**Fig. 4:**
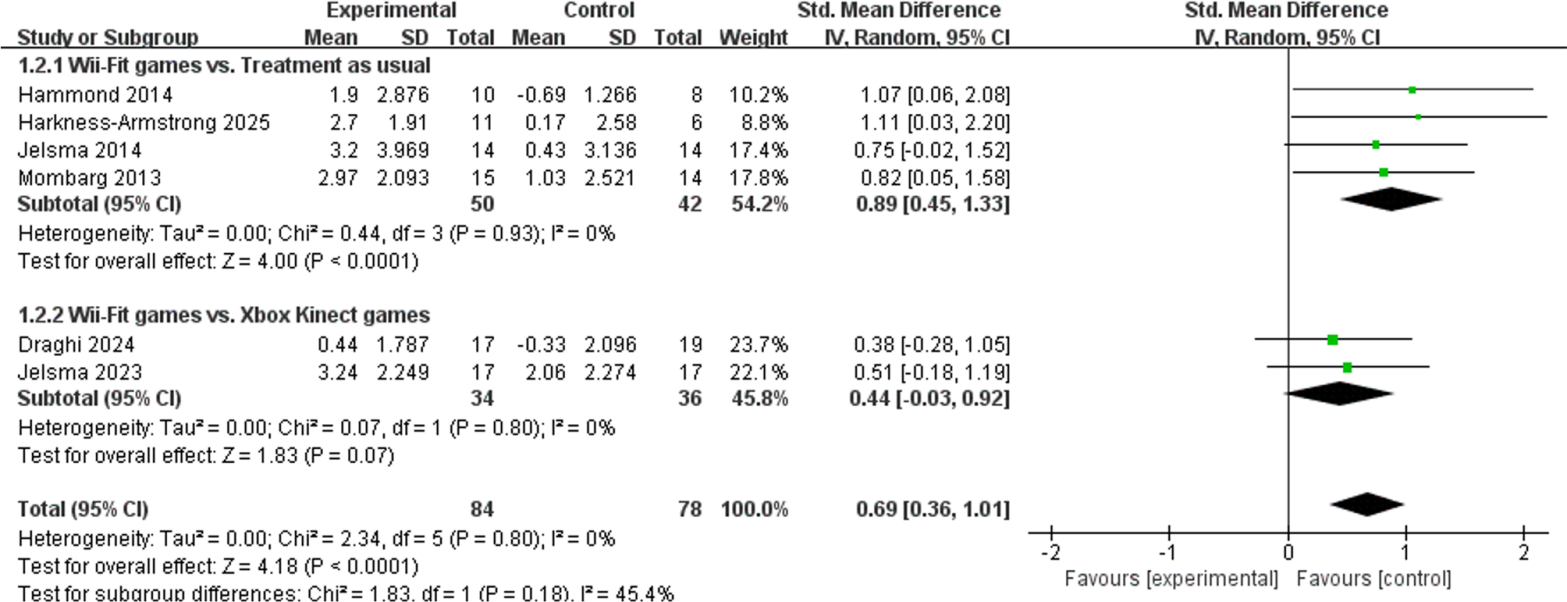
Forest plot of the effects of Wii-Fit game training on balance.

Note: Values to the right favor the physical exercise (experimental) group

Six studies (*n* = 162) evaluated the effect of Wii-Fit game training on balance ability in children with DCD [12, 15, 23–26]. As shown in Fig. 4, the meta-analysis showed statistically significant improvement in balance ability in the Wii-Fit group compared with the control group (SMD = 0.69, 95% CI [0.36, 1.01], *p* < 0.0001) with less heterogeneity (I²= 0%, *p* = 0.80). Agreement between studies was indicated. Subgroup analyses compared Wii-Fit game training under different control conditions: (1) Wii-Fit vs. Regular activities: this subgroup showed a large statistically significant effect in favor of Wii-Fit (SMD = 0.89, 95% CI [0.45, 1.33], *p* < 0.001), with no significant heterogeneity (I²= 0%, *p* = 0.93). (2) Wii-Fit vs. Xbox Kinect game training: There was no significant statistical difference between the two active interventions (SMD = 0.44, 95% CI [−0.03, 0.92], *p* = 0.07), nor was there significant heterogeneity (I²= 0%, *p* = 0.80). These results suggest that Wii-Fit game training significantly improved balance in children with DCD compared with regular activities. However, when compared directly with Xbox Kinect game training, Wii-Fit did not show a statistically significant advantage, suggesting comparable effects for both game-based interventions. Detailed records are shown in Table 4.

### Effects of Wii-Fit games training on agility in children with DCD

**Fig. 5:**
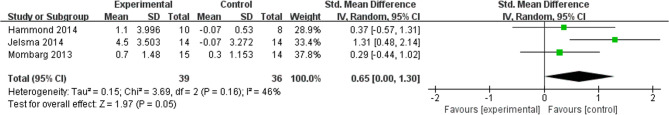
Forest plot of the effects of Wii-Fit game training on agility.

Note: Values to the right favor the physical exercise (experimental) group

Three studies (*n* = 75) evaluated the effects of Wii-Fit game training on agility in children with DCD [23, 25, 26]. As shown in Fig. 5, the meta-analysis revealed statistically significant improvements in agility compared with regular activities in the Wii-Fit group (SMD = 0.65, 95% CI [0.00, 1.30], *p* = 0.05), with less heterogeneity (I² = 46%,*p* = 0.16). The results showed that the Wii-Fit game training significantly improved agility in children with DCD compared to regular activities. Detailed records are shown in Table 4. Agility improvements were marginal (*p* = 0.05) and should be interpreted cautiously given the limited number of studies (*n* = 3).

### Publication bias and the analysis of sensitivity

Egger’s and Begg’s tests were used to assess publication bias in the outcome domain.

1. Motor skills: Begg’s test showed no significant bias (*p* = 0.308). However, Egger’s test suggested a small sample effect (*p* = 0.020). The funnel plot showed a slightly right-skewed distribution (Fig. 6).

**Fig. 6:**
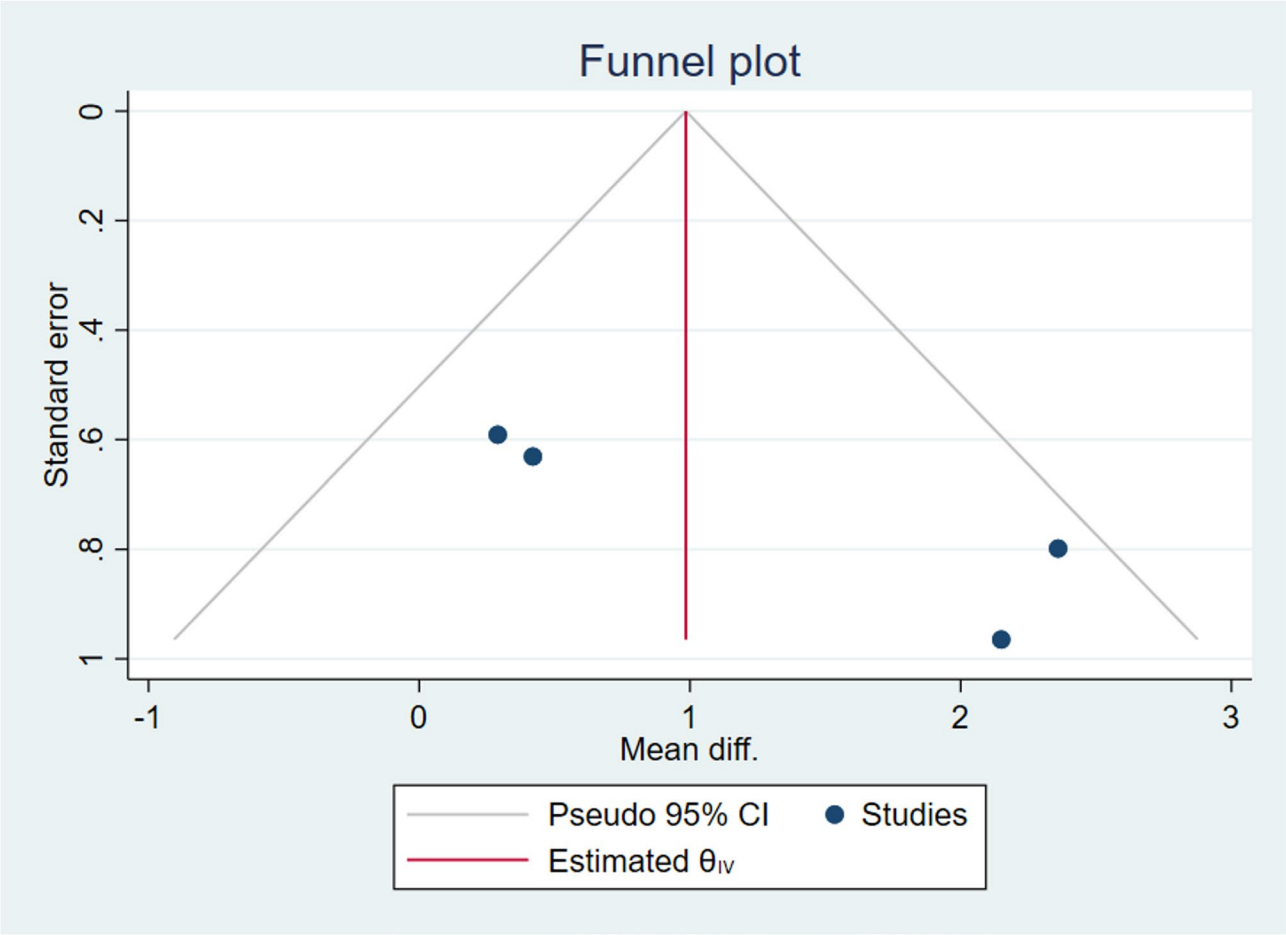
Funnel plot of the effect of Wii-Fit games training on motor skills
2. Balance: Egger’s test suggested a small sample effect (*p* = 0.047). Begg’s test was not significant (*p* = 0.060). The funnel plot showed a slight rightward bias (Fig. 7), consistent with Egger’s results.

**Fig. 7:**
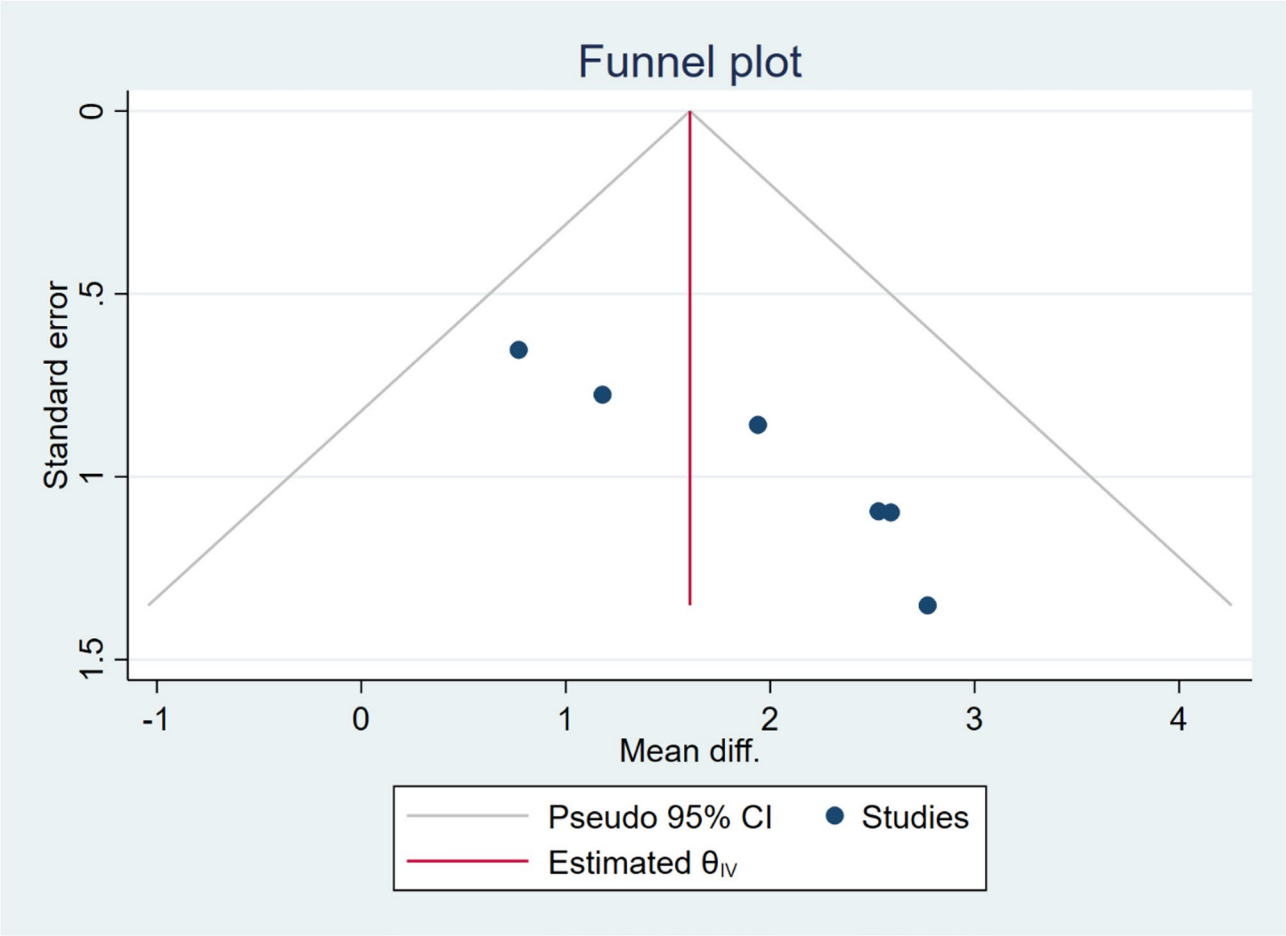
Funnel plot of the effect of Wii-Fit games training on balance
3. Agility: Egger’s test (*p* = 0.486) and Begg’s test (*p* = 1.000) showed no statistically significant bias. However, the funnel plot showed structural anomalies, which may be related to the small sample size (Fig. 8).

**Fig. 8:**
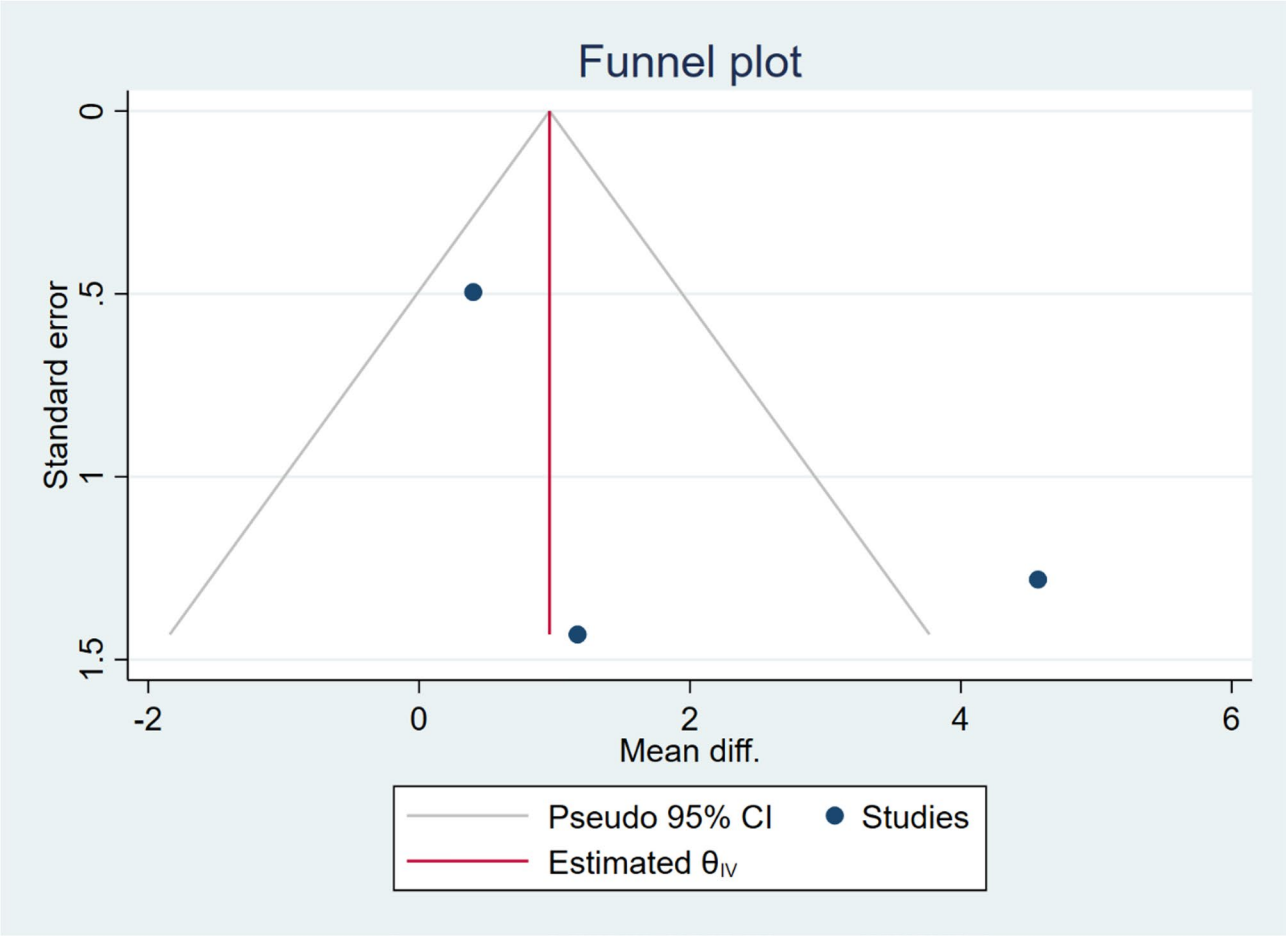
Funnel plot of the effect of Wii-Fit games training on agility

In conclusion, although Egger’s and Begg’s tests produced nonsignificant results (*p* > 0.05) and contradictory results, small sample sizes may have limited their power to detect actual publication bias. Therefore, these results should be interpreted with caution.

## Discussion

This meta-analysis comprehensively evaluated the therapeutic effects of Wii-Fit training across three domains: motor skills, balance, and agility. The results demonstrated that Wii-Fit training may lead to improvements in motor skills, notably enhancing fundamental movement capabilities. A statistically significant improvement in balance was also observed, consistent with clinical observations of enhanced balance function. While the effect size for agility improvement was comparatively smaller than the other two domains, it showed only a borderline significance.

The effect sizes observed for motor skills (SMD = 0.54) and balance (SMD = 0.69) fall within the moderate range, which indicates that the improvements may be of practical importance for children with DCD in real-world settings. By contrast, the effect for agility (SMD = 0.65, *p* = 0.05) should be regarded as tentative, as the limited number of studies and borderline statistical significance make its clinical relevance uncertain. These findings suggest that gamified intervention may be beneficial for children with DCD; however, they should be interpreted with caution because several included trials exhibited methodological limitations as identified in the ROB 2.0 assessment, and the GRADE evaluation rated the overall certainty of evidence as moderate.

The superiority of game-based training over traditional physical therapy in improving motor function lies in its engaging nature [27]. While traditional PT/OT may be perceived as less engaging by some children with DCD, effectiveness depends on task design and therapist interaction [28]. Game-based interventions like Wii-Fit may offer advantages in motivation due to intrinsic rewards, but well-designed conventional therapy can also achieve high compliance [2]. The enjoyable nature of these games motivates children to continue training, while the game challenges help them achieve the motor skill goals set by therapists [29]. Newer technologies, such as VR-based game therapy, can replace traditional motor skill training in terms of flexibility and interactivity [30], while providing comparable improvements in cardiopulmonary function [6]. However, the high equipment costs and complex operation procedures currently limit its widespread clinical application. Wii-Fit game training, with its low cost and high accessibility, is more suitable for home and community use [31]. This makes it particularly valuable for maintaining long-term participation in DCD treatment [15]. The functional improvements achieved through Wii-Fit and Kinect platforms demonstrate the broad clinical value of gamified therapy for DCD. Studies show that over 80% of DCD children maintain a strong interest and actively participate in Wii-Fit training [8], with better engagement leading to improved outcomes. Unlike traditional training, game-based therapy avoids training fatigue through constantly changing game scenarios and tasks - a crucial advantage for DCD children who require long-term rehabilitation [32].

The WHO guidelines recommend that children and adolescents should engage in at least 60 min of moderate to vigorous physical activity daily [33]. Wii-Fit and similar exergaming programs may contribute to meeting this requirement [34, 35]. Research has confirmed that Wii-Fit training can improve coordination, balance function, and cardiopulmonary capacity in children with DCD [2], aligning well with the WHO’s emphasis on aerobic exercise [6]. Nevertheless, the strength of this evidence is limited. Several included trials showed concerns in randomization, outcome reporting, and blinding, as highlighted in the ROB 2.0 evaluation, and the GRADE assessment downgraded the certainty of evidence to a moderate level. This indicates that further high-quality randomized trials are likely to impact the effect estimates and may alter current conclusions. Therefore, these findings should be regarded as preliminary and not definitive. Rehabilitation centers may consider adopting Wii-Fit training as a supplementary approach to traditional physical therapy. Comparative studies show that while Wii-Fit and Kinect produce similar improvements in balance and agility, Wii-Fit offers superior cost-effectiveness and greater accessibility [12]. Additionally, Wii-Fit training shows promise for inclusion in home-based rehabilitation programs. Digital exercise interventions, such as Wii Fit, provide patients with more engaging therapeutic options [36].

To ensure effective intervention outcomes, it is recommended that standardized training protocols be developed, specifying the frequency, duration, and selection of games for weekly sessions. Research shows that children with DCD demonstrate individualized learning curves with Wii-Fit interventions, highlighting the need for personalized training programs [16]. A comprehensive evaluation system should be established to assess both improvements in motor skills and children’s engagement levels. The focus should be on meaningful participation and enjoyment rather than simply completing interventions. This approach aligns with the WHO’s recommendation to cultivate lifelong physical activity habits from childhood [37].

### Limitations

Although this study provides substantial evidence supporting the application of Wii-Fit training for DCD rehabilitation, some limitations remain. While results suggest benefits, methodological limitations preclude definitive conclusions. First, the sample sizes in the included studies were relatively small, which may affect the accuracy of the study results. Agility improvements were marginal (*p* = 0.05) and should be interpreted cautiously given the limited number of studies (*n* = 3). Second, the intervention durations ranged only from 4 to 6 weeks in most studies, making it difficult to determine the optimal training period for the Wii-Fit intervention. While this meta-analysis primarily evaluates short-term effects (4–6 weeks), our discussion highlights the paucity of long-term follow-up data, underscoring the need for future trials with extended observation periods. Additionally, the included studies used different assessment tools and criteria to measure motor skills, balance, and agility, which may have affected the accuracy and comparability of the study results. Three studies were from the Netherlands (Jelsma et al., 2014; Jelsma et al.,2023; Mombarg et al., 2013), but each examined distinct cohorts and outcomes (balance vs. agility), minimizing sample duplication. These limitations reveal that future studies require larger sample sizes, more standardized experimental designs, and longer-term follow-up to comprehensively and systematically evaluate the clinical value of Wii-Fit training.

## Conclusion

This study suggests that Wii-Fit training may improve motor skills, balance, and agility in children with DCD. Future research should further explore its broader effects and the application of innovative techniques. In addition to motor function, the detection of cognitive benefits should be an important direction for future investigations.

## Supplementary Information

Supplementary Material 1

## Data Availability

The datasets used and analyzed during the current study are available from the first author (JC) on reasonable request.
